# Persisting Deficits in Health-Related Quality of Life of Colorectal Cancer Survivors 14–24 Years Post-Diagnosis: A Population-Based Study

**DOI:** 10.3390/curroncol30030257

**Published:** 2023-03-14

**Authors:** Melissa S. Y. Thong, Daniela Doege, Linda Weißer, Lena Koch-Gallenkamp, Lina Jansen, Heike Bertram, Andrea Eberle, Bernd Holleczek, Alice Nennecke, Annika Waldmann, Sylke Ruth Zeissig, Hermann Brenner, Volker Arndt

**Affiliations:** 1 Unit of Cancer Survivorship, Division of Clinical Epidemiology and Aging Research, German Cancer Research Center (DKFZ), 69120 Heidelberg, Germany; 2 Division of Clinical Epidemiology and Aging Research, German Cancer Research Center (DKFZ), 69120 Heidelberg, Germany; 3 Cancer Registry of North Rhine-Westphalia, 44801 Bochum, Germany; 4 Bremen Cancer Registry, Leibniz Institute for Prevention Research and Epidemiology—BIPS, 28359 Bremen, Germany; 5 Saarland Cancer Registry, 66024 Saarbrücken, Germany; 6 Hamburg Cancer Registry, 20539 Hamburg, Germany; 7 Institute for Social Medicine and Epidemiology, University of Lübeck, 23538 Lübeck, Germany; 8 Institute of Clinical Epidemiology and Biometry (ICE-B), Julius Maximilian University of Würzburg, 97080 Würzburg, Germany; 9 Cancer Registry of Rhineland-Palatinate, 55116 Mainz, Germany; 10 Division of Preventive Oncology, German Cancer Research Center (DKFZ), National Center for Tumor Diseases (NCT), 69120 Heidelberg, Germany; 11 German Cancer Consortium (DKTK), German Cancer Research Center (DKFZ), 69120 Heidelberg, Germany

**Keywords:** colorectal cancer, long-term survivors, health-related quality of life, population-based, non-cancer controls

## Abstract

(1) Background: The health-related quality of life (HRQOL) of colorectal cancer (CRC) survivors >10 years post-diagnosis is understudied. We aimed to compare the HRQOL of CRC survivors 14–24 years post-diagnosis to that of age- and sex-matched non-cancer controls, stratified by demographic and clinical factors. (2) Methods: We used data from 506 long-term CRC survivors and 1489 controls recruited from German population-based multi-regional studies. HRQOL was assessed with the European Organization for Research and Treatment of Cancer Quality of Life Core-30 (EORTC QLQ-C30) questionnaire. We estimated differences in the HRQOL of CRC survivors and controls with multiple regression, adjusted for age at survey, sex, and education, where appropriate. (3) Results: CRC survivors reported poorer social functioning but better health status/QOL than controls. CRC survivors, in general, had higher levels of symptom burden, and in particular diarrhea and constipation, regardless of demographic or clinical factors. In stratified analyses, HRQOL differed by age, sex, cancer type, and having a permanent stoma. (4) Conclusions: Although CRC survivors may have a comparable health status/QOL to controls 14–24 years after diagnosis, they still live with persistent bowel dysfunction that can negatively impact aspects of functioning. Healthcare providers should provide timely and adapted follow-up care to ameliorate potential long-term suffering.

## 1. Introduction

Earlier detection and better treatment have improved the relative 5-year survival after colorectal cancer (CRC) [1,2,3,4], even among survivors diagnosed at an older age [5]. Among stage I–III CRC survivors, minimal excess mortality for colon cancer may be achieved within six years post-diagnosis, and nine years post-diagnosis for rectal cancer [6]. 

Besides survival, health-related quality of life (HRQOL) is now considered an important outcome of cancer treatments [7]. For most CRC survivors, treatment side effects such as pain or fatigue are acute and impairments do recede back to pre-treatment levels, with concurrent improvements in HRQOL [8]. Therefore, the majority of CRC survivors can expect to live a large proportion of their remaining life in relatively good health [9]. Nevertheless, a significant proportion of survivors continue to struggle with the sequelae of CRC; for example, treatment-related symptoms that can negatively influence aspects of HRQOL years after diagnosis [10,11]. 

In our previous study on long-term CRC survivors 5–16 years post-diagnosis, we noted that survivors reported a global health/quality of life (QOL) comparable to that of age-matched non-cancer controls [12]. Nevertheless, specific problems with constipation and diarrhea persist, and younger (<65 years) CRC survivors reported lower levels of role and social functioning, and higher levels of financial problems. 

There is a paucity of research on the HRQOL of CRC survivors extending >10 years post-diagnosis [13,14,15,16]. We recently reported that the overall global health/QOL of survivors of colorectal, breast, and prostate cancers 14–24 years after diagnosis was comparable to that of non-cancer controls [17]. The current study builds on that publication with a focus on CRC survivors and more in-depth analyses of the associations of factors such as age at survey and cancer type (colon and rectal) on HRQOL of CRC survivors in comparison to a matched non-cancer population.

Our main study objectives were to compare the HRQOL of CRC survivors 14–24 years post-diagnosis to that of non-cancer controls. Furthermore, we were interested in whether HRQOL was associated with demographic (age at survey, sex, education) and clinical (cancer type, treatment, disease status) factors.

## 2. Materials and Methods

### 2.1. CAESAR Study

The population-based CAncEr Survivorship—A multi-Regional (CAESAR) study aimed to describe the long-term HRQOL of colorectal, breast, and prostate cancer survivors. The German Cancer Research Center (Deutsches Krebsforschungszentrum, DKFZ) conducted the study in collaboration with six epidemiologic cancer registries in Germany (Bremen, Hamburg, North Rhine-Westphalia, Rhineland-Palatinate, Schleswig-Holstein, and Saarland). This study used data from five cancer registries as no CRC survivors were recruited from Schleswig-Holstein for logistical reasons. Cancer survivors diagnosed between January 1994 and June 2004, as registered in the participating cancer registries, and aged between 20 and 75 years at diagnosis, were eligible. Details of the initial study have been described elsewhere [18]. Initial recruitment was in 2008–2010, 5–16 years post-diagnosis. Between 2018 and 2019, a follow-up assessment was conducted among survivors (14–24 years post-diagnosis) who had given consent at initial recruitment to be re-contacted and who were still alive [17]. Of the 2704 (62.9%) participants who returned a full-length questionnaire at follow-up assessment, 506 (19%) were CRC survivors. Further details about the response have been previously reported, namely that CRC survivors were less likely to participate at follow-up in contrast to breast or prostate cancer survivors [17].

### 2.2. LinDe Study

An individual level HRQOL from a representative sample of the German population was accessed from the Lebensqualität in DEutschland (‘Quality of life in Germany’, LinDE) study [18]. Eligible participants aged 18 and above, stratified by age and sex, were randomly selected from the general German population via regional municipal offices. Data collection was conducted between 2013 and 2014. Potential participants received detailed study information and a questionnaire by mail. Non-respondents received two follow-up reminder mails and a telephone contact (or one mailed reminder or home visit, if necessary). Further details of sample selection are reported elsewhere [18]. For the current study, we selected as controls those participants who completed a full questionnaire, were cancer-free, and of comparable age to the CRC sample at CAESAR follow-up.

### 2.3. HRQOL Assessment

HRQOL and financial difficulty were assessed with the European Organization for Research and Treatment of Cancer Quality of Life Core-30 (EORTC QLQ-C30) questionnaire [19]. This 30-item questionnaire consists of five functional scales (physical, role, cognitive, emotional, social), a global health/QOL scale, and nine items/scales on symptoms and financial impact. Answers are ranged from 1 (not at all) to 4 (very much), and from 1 (‘very poor’) to 7 (‘excellent’) for items in the global health/QOL scale. All raw scores were linearly transformed to scales of 0–100 using standard procedures [20]. Higher functioning and global health/QOL scores indicate better function or health status; higher scores on symptoms and financial difficulty indicate greater symptom burden and financial problems. 

### 2.4. Demographics and Clinical Data

The CAESAR questionnaire contained questions concerning sociodemographic factors and clinical history. Self-reported data include primary treatment received, disease recurrence since index cancer (recurrence, metastasis, new primary cancer), and comorbid conditions. Participating cancer registries provided clinical data such as date of diagnosis and the tumor stage (from the initial 2008–2010 data collection). Other self-reported data collected in the initial data survey include monthly household income, education, type of employment (e.g., employee, self-employed), and work situation (e.g., full/part-time). The classification of cancer site was according to the International Classification of Diseases-10 codes. 

Relevant data from surveys in 2008–2010 and 2018–2019 were combined for this analysis.

### 2.5. Statistical Analyses

We compared sociodemographic differences between CRC survivors and population controls with Cochran–Mantel–Haenszel tests. Although the age distribution of the population controls reflected a stratified sampling scheme, controls were significantly younger than CRC survivors. Therefore, we used direct standardization for further comparisons of sample characteristics, using the age and sex distribution of CRC survivors as standard.

We used multiple linear regression to derive least square mean HRQOL scores of CRC survivors and controls. In models stratified by disease status, we categorized CRC survivors accordingly: stage I–III at diagnosis and with no reports of recurrence/metastasis/new cancer at follow-up (‘disease-free’); stage IV at diagnosis or self-report of subsequent recurrence/metastasis/new cancer after CRC diagnosis (’active disease’). Linear regression models were adjusted for age (in 5-year bands: 45–49, 50–54…90–94), sex, and education at the time of the survey, where appropriate. Covariates included for adjustment in the models were selected a priori, based on previous associations with HRQOL reported in the literature [21,22]. Although employment, relationship status, and comorbidities differed between survivors and controls, we did not include these as covariates in the models because they may reflect the situation at the time of survey. Potential differences in those variables between CRC survivors could be a consequence of the cancer. 

Missing data were imputed using the Markov chain Monte Carlo method with 25 repetitions to reduce possible bias due to missing values (generally <10%). All analyses were conducted with SAS (version 9.4 for Windows, SAS Institute Inc., Cary, NC, USA). Statistical significance is determined at *p* < 0.05 (two-sided). The *p*-values were not adjusted for multiple testing, referring to the individual tests rather than a global test for differences.

## 3. Results

### 3.1. Non-Response Analysis

**Figure 1:**
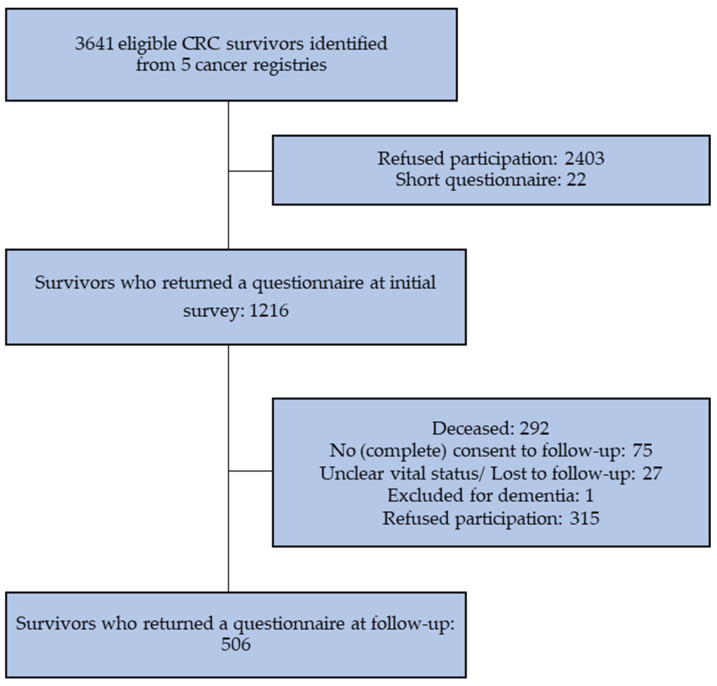
Flowchart of inclusion of CRC survivors.

Of the 1216 CRC participants in the initial CAESAR survey, 292 (24%) had died before follow-up. Of the survivors eligible for the follow-up survey (*n* = 924), 506 (55%) returned a completed questionnaire (Figure 1). 

Respondents to the follow-up survey were younger at initial survey, more likely to be male, and to be treated surgically (Table 1). Non-respondents were more likely to be diagnosed with colon cancer and less likely to have received chemotherapy or radiotherapy. Those who had died before follow-up were older at time of diagnosis, were more often male, had stage III or IV CRC, and had a permanent stoma. 

### 3.2. Characteristics of CRC Survivors and Controls

CRC survivors were older and more likely to be male, when compared with the controls (Table 2). Even after age and sex standardization, there remained significant differences in the characteristics between the two groups. CRC survivors were less likely to be employed, more likely to be in a partnered relationship, and generally less likely to have cardiovascular, gastro-intestinal, or neuro-skeletal comorbidities.

### 3.3. Characteristics of CRC Survivors by Cancer Type

There were no significant differences in characteristics between colon and rectal cancer survivors except for treatment. Rectal cancer survivors were more likely to have received chemotherapy and radiotherapy, and to have a permanent stoma (Table 3).

### 3.4. HRQOL of CRC Survivors and Controls

**Figure 2:**
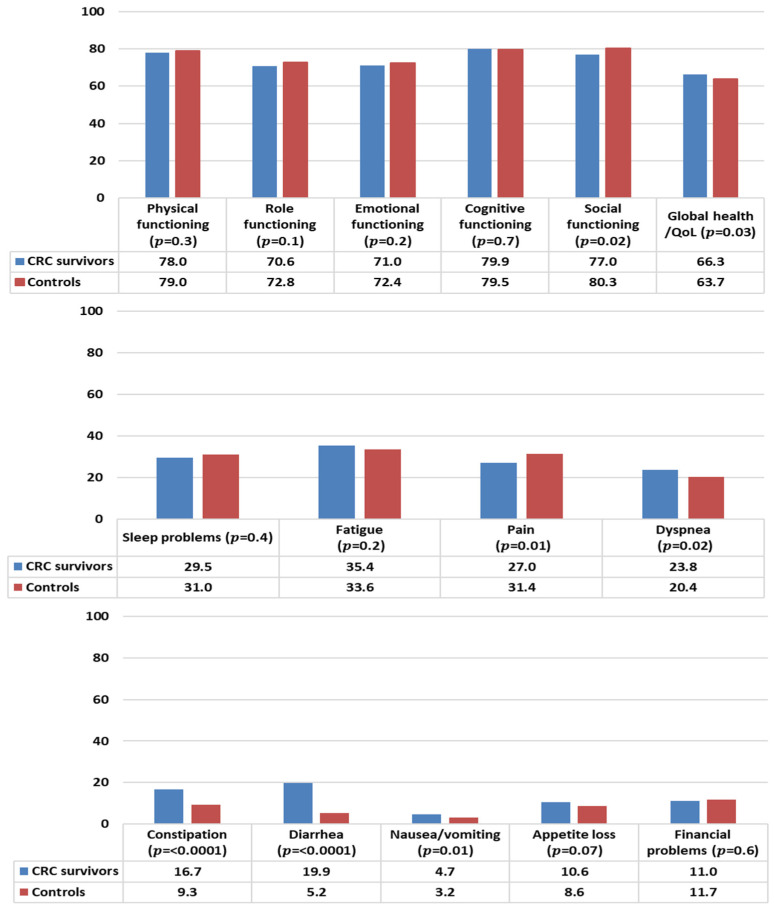
Mean EORTC QLQ-C30 scores of colorectal cancer (CRC) survivors and controls. Models are adjusted for age at survey, sex, and education. All results are based on 25 imputations of missing values.

In general, CRC survivors reported levels of functioning comparable to controls, except for social functioning that was statistically significantly lower than controls (Figure 2). CRC survivors reported a better health status/QOL than controls. With respect to symptom burden, CRC survivors reported less pain but more problems with dyspnea, constipation, diarrhea, and nausea/vomiting than controls. 

### 3.5. HRQOL of CRC Survivors and Controls, Stratified by Demographic Factors

#### 3.5.1. By Age at Survey

**Figure 3:**
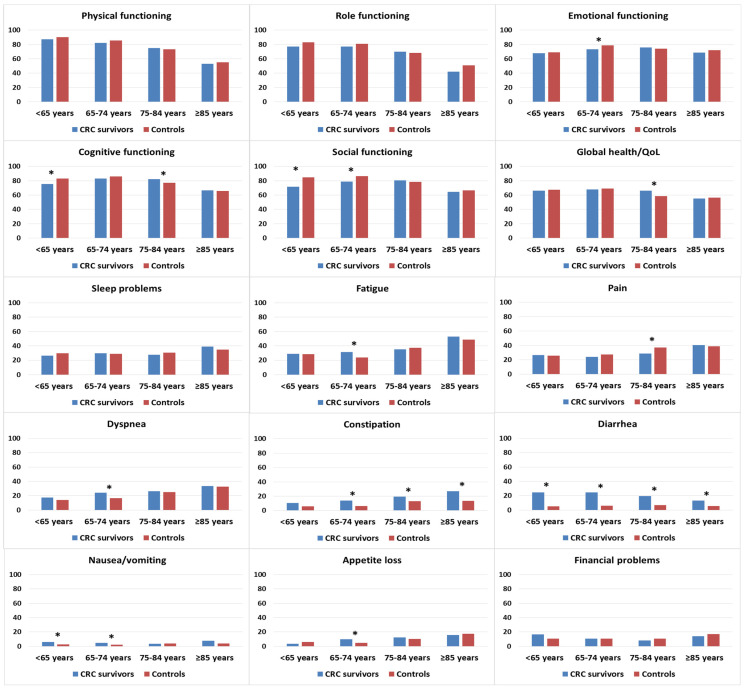
Mean EORTC QLQ-C30 scores of colorectal cancer (CRC) survivors and controls, stratified by age at survey. Models are adjusted for age at survey, sex, and education. Asterisks (*) indicate significantly differences (*p* < 0.05) between the subgroups. All results are based on 25 imputations of missing values.

CRC survivors who were <75 years old tended to report lower functioning scores than age-matched controls, although significant differences were noted only in emotional functioning, cognitive functioning, and social functioning (Figure 3). Survivors who were 75–84 years old reported better cognitive functioning and global health/QOL than controls in the same age group. In terms of symptom burden, CRC survivors in the 65–74 years age group reported higher levels of fatigue, dyspnea, and appetite loss than age-matched controls. Survivors in the age groups < 65 and 65–74 years were more likely to report higher levels of nausea/vomiting than controls in similar age groups. Regardless of age, CRC survivors reported higher levels of constipation and diarrhea. 

#### 3.5.2. By Education

**Figure 4:**
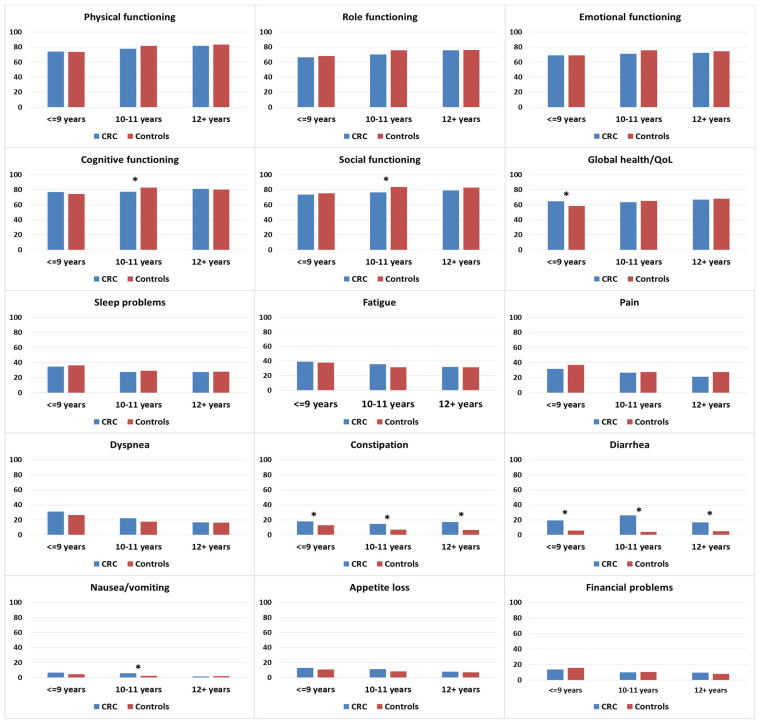
Mean EORTC QLQ-C30 scores of colorectal cancer (CRC survivors) and controls, stratified by education. Models are adjusted for age at survey and sex. Asterisks (*) indicate significantly significant differences (*p* < 0.05) between the subgroups. All results are based on 25 imputations of missing values.

There were no differences in functioning when stratified by years of education, with the exception of cognitive and social functioning. In these two subscales, CRC survivors with 10–11 years of education reported lower scores than controls with equivalent education (Figure 4). Among those with ≤9 years of education, CRC survivors reported higher levels of global health/QOL than controls. For symptom burden, CRC survivors reported significantly more problems with constipation and diarrhea than controls, irrespective of education level. Increased problems with nausea/vomiting in CRC survivors compared to controls were observed in the group with 10–11 years of education.

### 3.6. HRQOL of Survivors by Cancer Type and Controls, Stratified by Sex

**Figure 5:**
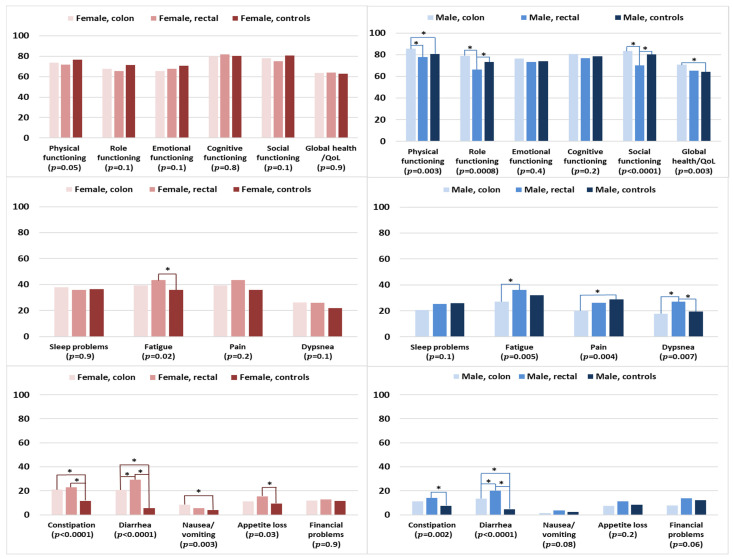
Mean EORTC QLQ-C30 scores of survivors (colon and rectal cancer) and controls, stratified by sex. Models are adjusted for age at survey and education. The *p*-values shown indicate the global comparison between cancer survivors and controls, separated by sex. The line spans and asterisks (*) indicate the subgroups that showed significant differences in pairwise comparison. For example, the line span for physical functioning in male colon survivors is across three columns, indicating that male colon survivors reported significantly higher physical functioning when compared with male controls. All results are based on 25 imputations of missing values.

The functioning and global health/QOL scores of female colon and rectal cancer survivors were comparable with those of female controls (Figure 5). Female rectal cancer survivors were more likely to be fatigued and have appetite loss when compared with female controls. Female colon cancer survivors experienced more problems with nausea/vomiting when compared with female controls. Female colon and rectal cancer survivors reported significantly more problems with constipation than female controls. For diarrhea, female rectal cancer survivors reported significantly higher levels than female colon cancer survivors and controls.

Among the male participants, rectal cancer survivors reported the lowest levels of functioning, namely in the domains of physical functioning (versus colon cancer survivors), and role and social functioning in comparison with colon cancer survivors and controls (Figure 5). In contrast, male colon cancer survivors reported better physical functioning and global health/QOL than male controls. Male rectal cancer survivors tended to report higher levels of symptom burden than male colon survivors or controls, notably for fatigue, dyspnea, constipation, and diarrhea. Male colon cancer survivors reported lower levels of pain but higher levels of diarrhea when compared with male controls.

### 3.7. HRQOL of Survivors Stratified by Clinical Factors, and Controls

#### 3.7.1. By Stoma Status

**Figure 6:**
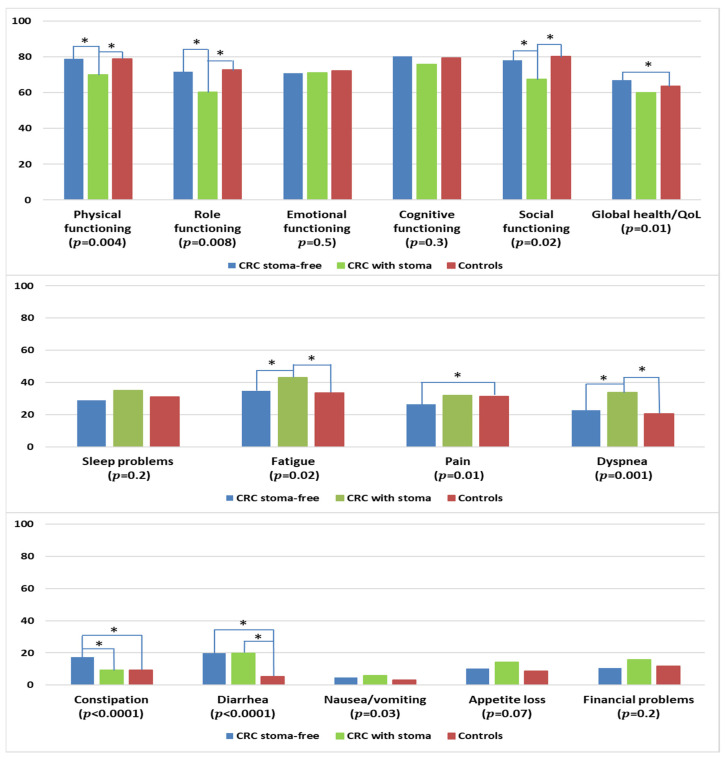
Mean EORTC QLQ-C30 scores of colorectal cancer (CRC) survivors with and without permanent stoma, and controls. Models are adjusted for age at survey, sex, and education. The *p*-values shown indicate the global comparison between cancer survivors with and without stoma and controls. The line spans and asterisks (*) indicate the subgroups that showed significant differences in pairwise comparison. For example, the line span for global health/QOL is across three columns, indicating that stoma-free CRC survivors reported significantly higher global health/QOL when compared with controls. All results are based on 25 imputations of missing values.

CRC survivors with a permanent stoma reported significantly lower levels of physical, role, and social functioning when compared with stoma-free CRC survivors or controls (Figure 6). Stoma-free survivors reported better global health/QOL than controls. CRC survivors with a stoma were more likely to report higher levels of fatigue and dyspnea when compared with colon cancer survivors and controls. Stoma-free CRC survivors reported less pain than controls but higher levels of constipation, in comparison with CRC survivors with permanent stoma or controls. CRC survivors, regardless of stoma status, reported more problems with diarrhea than controls. 

#### 3.7.2. By Disease Status

**Figure 7:**
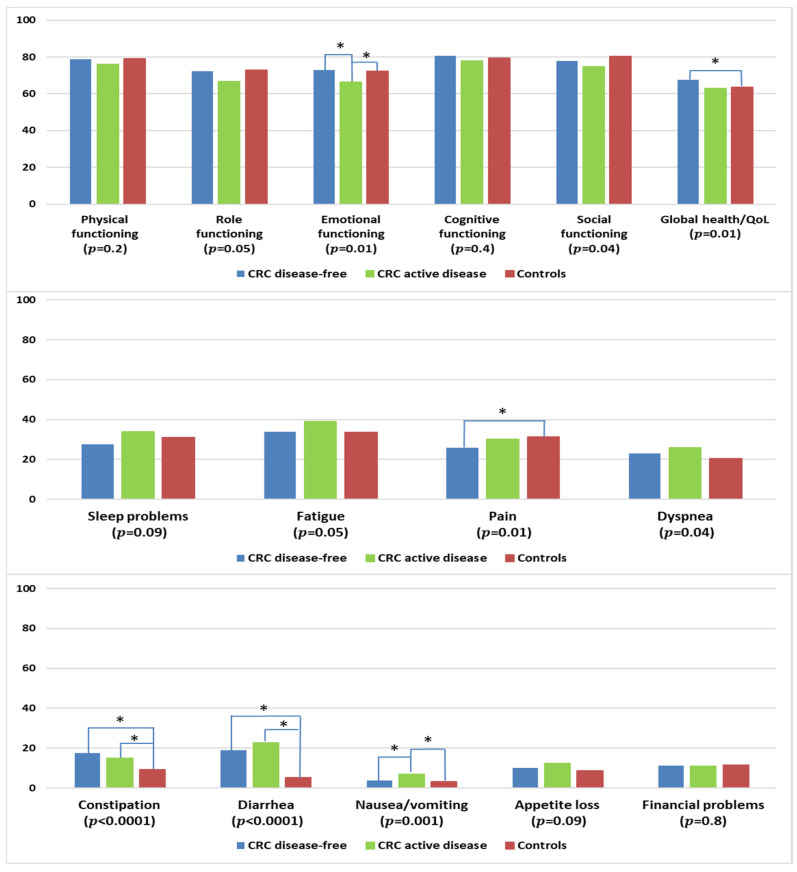
Mean EORTC QLQ-C30 scores of colorectal cancer (CRC) survivors by disease status, and controls. Models are adjusted for age at survey, sex, and education. Disease-free: stage I–III at diagnosis and remaining disease-free at follow-up according to self-report; active disease: stage IV at diagnosis or reported subsequent recurrence, metastasis, or primary cancer. The *p*-values shown indicate the global comparison between cancer survivors with and without active disease and controls. The line spans and asterisks (*) indicate the subgroups that showed significant differences in pairwise comparison. For example, the line span for global health/QOL is across three columns, indicating that disease-free CRC survivors reported significantly higher global health/QOL when compared with controls. All results are based on 25 imputations of missing values.

CRC survivors with active disease reported lower levels of emotional functioning when compared with disease-free survivors or controls (Figure 7). Survivors who remained disease-free reported higher levels of global health/QOL and lower levels of pain than controls. CRC survivors, regardless of disease status, had more problems with constipation and diarrhea than controls. Survivors with active disease reported more problems with nausea/vomiting than disease-free survivors or controls.

## 4. Discussion

With CRC survivors living longer, it is important to have a better understanding of the quality of this prolonged survival. We found that CRC survivors 14–24 years after diagnosis, in general, had comparable levels of functioning and global health/QOL to age- and sex-matched population controls. Nevertheless, we see persistent deficits in aspects of functioning and a higher symptom burden in subgroup analyses. 

We observed a consistent pattern of lower levels of social functioning among CRC survivors, most prominent among survivors who were <75 years old, with 10–11 years of education, male rectal cancer survivors, and survivors with a permanent stoma. In addition, constipation and diarrhea remain as problems of significance among survivors in comparison with controls, irrespective of age, sex, education, cancer type, and disease status. Treatments for CRC have been associated with chronic bowel dysfunction [11,23], of which the impact on HRQOL is significant [24]. It is intuitive that persistent bowel problems could hinder daily activities and restrict social participation [25]. CRC survivors described feelings of embarrassment and a loss of control resulting from bowel dysfunction, and struggles to self-manage these problems, e.g., toilet mapping, extra planning, or diet changes [26,27].

Male rectal cancer survivors and survivors with permanent stoma reported similar patterns of deficits, namely poorer levels of physical, role, and social functioning, and higher levels of fatigue, dyspnea, and diarrhea than comparison groups. These results are consistent with concerns commonly shared by individuals living with a stoma [28]. Problems associated with a stoma are well documented [29,30], and can pose a threat to body image and self-confidence [25]. A stoma can restrict physical, role (including work) and social functioning [25], and reduce HRQOL [31]. 

Survivors younger than 75 years at survey were more likely to have deficits in emotional, cognitive, and social functioning. Keeping in mind that our study involved survivors who were diagnosed up to 24 years previously, this would suggest that poorer emotional and social functioning scores observed closer to the time since diagnosis can persist in survivors who were diagnosed at a younger age [32]. Furthermore, in the study by Dunn et al., survivors who were diagnosed at younger age not only had consistently poorer emotional and social functioning up to 5 years post-diagnosis, but reported consistently lower life satisfaction [32]. With a trend in increasing incidence of CRC among persons younger than 50 years of age [33], these results indicate that greater attention to the adaption process of life after cancer for younger CRC survivors is paramount. A better identification of vulnerable survivors and the timely addressing, e.g., closer to time since diagnosis, of potential physical, psychosocial, and financial needs of young-onset CRC survivors may reduce the long-term burden of CRC. 

### 4.1. Clinical Implications

Despite the improved prognosis after CRC diagnosis, a significant proportion of survivors are living with long-term symptoms and functioning impairments. Existing clinical guidelines on CRC follow-up care tend to focus on the detection of early recurrences, the timing of follow-ups, and appropriate diagnostic tools, but few guidelines provide adequate recommendations for the management of long-term symptoms [34]. For example, the German guideline provides recommendations that CRC survivors should be encouraged to improve their health and QOL. However, the guideline does not give specifics on how or from whom this encouragement should be provided, but states that survivors benefit if they self-manage their symptoms and side effects [35]. Bowel dysfunction remains a significant problem for CRC survivors. Although there are treatments for bowel problems including recommendations of diet adjustments [36,37,38], survivors may not be aware of such help. There is a discrepancy between healthcare professionals reporting the provision of dietary advice and the proportion of survivors remembering that such advice was provided [39]. Studies have shown that survivors respond better to clinician-provided recommendations for lifestyle change [40], and are positive about the multidisciplinary approach to symptom management adopted by survivorship care clinics [41]. Changes in diagnostics and treatments for CRC in recent years (e.g., the advent of minimally invasive surgery) [42,43], and increasing awareness of unmet needs, such as the provision of psycho-oncological support could have improved the HRQOL of survivors with a more recent diagnosis [44,45]. However, a significant proportion of long-term survivors may not have benefitted from these recent improvements in survivorship care [46]. Reasons could include an unawareness of the potential persisting or late effects of treatment and help thereof in survivors and clinicians, was ‘lost’ to follow-up when transitioning from acute to long-term survivorship or fragmented delivery of health care. Collectively, these results suggest that clinical guidelines need to be updated by incorporating emerging evidence on supportive care interventions, to include more comprehensive recommendations for the management of the long-term symptoms of CRC survivors [34]. Recently, the European Society for Medical Oncology (ESMO) published an Expert Consensus Statement on Cancer Survivorship, highlighting the need for the provision of care plans for survivors to monitor and manage the physical effects of cancer [47]. Recommendations include the provision of early (during active treatment phase) educational and self-management support to anticipate possible late or persistent survivorship care needs. Furthermore, at-risk survivors could benefit from a coordinated multidisciplinary approach to symptom management, from earlier detection to established referral pathways to relevant specialists [47,48].

### 4.2. Strengths and Limitations

Our population-based study focused on the long-term HRQOL of CRC survivors 14–24 years after diagnosis, a group that is currently understudied. Another strength is the adequate sample size that allowed for subgroup analyses. However, our study has several limitations to consider. Although our sample size is adequate, nevertheless with a response rate of 55%, our results may not be generalizable to CRC survivors of a similar vintage. There were significant differences in characteristics between respondents and non-respondents. This potential participation bias may influence the reported mean HRQOL estimates. Participants provided clinical data in the context of the initial study round in 2008–2010. As some participants were diagnosed up to 16 years before the initial survey, the possibility of under-reporting or problem with recall (e.g., wrong time frame, misclassification of endoscopic or laparoscopic surgery) has to be discussed. It is unlikely that almost 20% of all participants did not undergo surgery for primary treatment. In addition, the participants who were classified as stoma-free could have had a temporary stoma although we do not know the duration and when the stoma was removed. We reported on the cross-sectional associations of clinical and demographic factors with HRQOL, and therefore the results should not be interpreted as causal associations. 

## 5. Conclusions

In conclusion, while CRC survivors 14–24 years after diagnosis may have a comparable health status/QOL to age- and sex-matched controls, they still live with persistent bowel dysfunction that can negatively impact aspects of functioning. Health care providers should regularly screen for potential unmet needs and provide timely follow-up care that is adapted to the needs of CRC survivors, to ameliorate potential long-term suffering. 

## Figures and Tables

**Table 1:** Characteristics of sample according to response to follow-up survey.

*n* (%)	Respondents (*n* = 506)	Non- Respondents (*n* = 418)	Died (*n* = 292)	*p*-Value
**Mean age at initial survey ± SD**	67.8 ± 8.4	71.2 ± 8.5	73.7 ± 7.9	**<0.0001**
**Time to initial survey since diagnosis ± SD**	8.4 ± 2.5	8.5 ± 2.5	8.8 ± 2.5	0.1
**Sex**				**<0.0001**
Female	207 (41)	208 (50)	89 (30)	
Male	300 (59)	210 (50)	203 (70)	
**Tumor type**				**0.0009**
Colon	280 (55)	275 (66)	157 (54)	
Rectal	227 (45)	143 834)	135 (46)	
**Tumor stage**				**0.03**
I	123 (24)	103 (25)	54 (18)	
II	145 (29)	113 (27)	80 (27)	
III	134 (26)	94 (22)	83 (28)	
IV	16 (3)	18 (4)	22 (8)	
Missing	89 (18)	90 (22)	53 (18)	
**Surgery**				**0.003**
Yes	408 (80)	293 (70)	207 (71)	
No	90 (18)	105 (25)	75 (26)	
Missing	9 (2)	16 (5)	10 (3)	
**Chemotherapy**				**0.01**
Yes	235 (46)	163 (39)	146 (50)	
No	269 (51)	239 (57)	137 (47)	
Missing	13 (3)	16 (4)	9 (3)	
**Radiotherapy**				**0.01**
Yes	117 (23)	68 (16)	66 (23)	
No	360 (71)	321 (77)	193 (66)	
Missing	30 (6)	29 (7)	33 (11)	
**Immuno-/antibody therapy**				0.6
Yes	19 (4)	12 (3)	12 (4)	
No	451 (89)	368 (88)	249 (85)	
Missing	37 (7)	38 (9)	31 (11)	
**Permanent stoma**				**<0.0001**
Yes	55 (11)	35 (8)	59 (20)	
No	452 (89)	382 (92)	233 (80)	
Missing	0	1 (0.2)	0	

Percentages may not add up to 100% due to rounding up. Statistically significant *p*-values are highlighted in bold (*p* < 0.05).

**Table 2:** Characteristics of CRC survivors and population controls.

	CRC Survivors		Population Controls			*P*_crude_ (χ^2^)	*P*_adjusted_ (CMH) ^a^
	*n*	%	*n*	%	%adj ^a^		
**Total**	506	100.0	1489	100.0			
**Age at survey**						**<0.0001**	-
<65 years	52	10.3	665	44.7	10.3		
65–74 years	118	23.3	401	26.9	23.3		
75–84 years	268	53.0	317	21.3	53.0		
≥85 years	68	13.4	106	7.1	13.4		
**Mean age at survey** **(SD)**	76.1	(8.4)	67.0	(11.8)			
**Sex:** Male	299	59.1	717	48.2	59.1	**<0.0001**	-
**Education**						**0.0006**	0.61
≤9 years	264	52.1	631	42.4	51.8		
10–11 years	111	21.9	374	25.1	19.3		
≥12 years	131	25.9	484	32.5	28.9		
**Employment at survey**						**<0.0001**	**<0.0001**
Full-time	19	3.8	315	21.1	5.5		
Part-time	15	3.0	169	11.4	3.5		
Early retired/unemployed	394	77.9	790	53.1	75.9		
Houseman/wife	49	9.6	157	10.5	11.3		
Other	29	5.7	58	3.9	3.8		
**In partnered relationship**	390	77.0	1104	74.2	68.5	0.20	**0.0002**
**Self-reported comorbidities**							
Stroke	30	5.9	71	4.8	7.2	0.3	0.3
Myocardial infarction	39	7.6	70	4.7	7.5	**0.01**	0.9
Angina pectoris	43	8.5	152	10.2	16.4	0.2	**<0.0001**
Chronic heart failure	63	12.4	168	11.3	19.4	0.4	**0.002**
Neurological disease	13	2.6	66	4.4	6.0	0.06	**0.006**
Upper gastrointestinal problems	51	10.1	193	13.0	14.1	0.08	**0.04**
Arthrosis	202	39.9	525	35.3	40.0	0.06	0.9
Rheumatism/arthritis	55	10.8	233	15.6	20.7	**0.007**	**<0.0001**
Osteoporosis	69	13.7	167	11.2	16.5	0.1	0.2
Diabetes mellitus	76	15.1	216	14.5	18.9	0.7	0.07
Chronic obstructive pulmonary disorder	35	6.8	58	3.9	4.8	**0.007**	0.1
Asthma	32	6.3	89	6.0	5.5	0.7	0.6
Hearing problems	150	29.5	301	20.2	32.7	**<0.0001**	0.2
Chronic back pain	187	37.0	522	35.0	38.3	0.4	0.6
Depression (ever)	53	10.5	242	16.2	13.4	**0.001**	0.1
Anxiety/panic attack (ever)	25	5.0	139	9.3	7.0	**0.002**	0.1
**Number of comorbidities**						**0.01**	**0.001**
None	115	22.8	367	24.6	15.3		
One	101	20.0	373	25.0	22.7		
Two to three	173	34.1	412	27.7	32.1		
Four or more	116	23.0	337	22.7	29.9		

^a^ Rates of population controls are standardized by the age and sex distribution of the cancer survivor cohort. CMH: Cochran–Mantel–Haenszel tests; χ^2^: chi-squared tests. All results are based on 25 imputations of missing values. Numbers might not add up to the total sample size due to rounding up of imputed results. Percentages might not add up to 100% due to rounding up. Statistically significant *p*-values are highlighted in bold (*p* < 0.05).

**Table 3:** Characteristics of cancer survivors by cancer type.

	Colon (*n* = 279)		Rectal (*n* = 227)		*p*-Value ^b^
	*n*	% MI ^a^	*n*	% MI ^a^	
**Sex**					0.2
Female	121	43.4	86	37.9	
Male	158	56.6	141	62.1	
**Age at survey**					0.9
<65 years	27	9.7	25	11.0	
65–74 years	64	22.9	54	23.8	
75–84 years	149	53.4	119	52.4	
≥85 years	39	14.0	29	12.8	
**Mean age at survey** **(SD)**	76.3	(8.6)	75.8	(8.1)	0.5
**Mean time since diagnosis**	16.7	(2.6)	16.8	(2.5)	0.8
**Cancer stage at diagnosis (UICC)**					0.1
I	70	25.2	73	32.2	
II	112	40.0	72	31.8	
III	83	29.7	74	32.8	
IV	14	5.1	7	3.3	
**Primary treatment**					
Surgery	221	79.4	193	85.2	0.09
Chemotherapy	120	42.9	121	53.2	**0.02**
Radiotherapy	23	8.2	103	45.5	**<0.0001**
Immuno-/antibody therapy	5	5.2	5	2.3	0.09
**Permanent stoma**	2	0.7	53	23.3	**<0.0001**
**Disease progression**					0.7
None	208	74.6	170	74.9	
Between diagnosis and 1st survey	30	10.8	20	8.8	
Between 1st and 2nd survey	41	14.7	37	16.3	
**Number of comorbidities**					0.7
None	60	21.6	55	24.3	
One	60	21.6	41	18.1	
Two to three	96	34.3	77	34.0	
Four or more	63	22.5	54	23.6	

Percentages might not add up to 100% due to rounding up. ^a^ MI: based on 25 imputations. ^b^ *p*-values of chi-squared tests. UICC: Union for International Cancer Control classification. Statistically significant *p*-values are highlighted in bold (*p* < 0.05).

## Data Availability

Data is available from the authors upon reasonable request.
